# Effectiveness of companion-intensive multi-aspect weight management in Chinese adults with obesity: a 6-month multicenter randomized clinical trial

**DOI:** 10.1186/s12986-020-00511-6

**Published:** 2021-02-03

**Authors:** Wanzi Jiang, Shushu Huang, Shuai Ma, Yingyun Gong, Zhenzhen Fu, Li Zhou, Wen Hu, Guofang Mao, Zhimin Ma, Ling Yang, Guangfeng Tang, Xiaofang Sun, Ping Zhang, Jianling Bai, Lei Chen, Bimin Shi, Xinhua Ye, Hongwen Zhou

**Affiliations:** 1 Department of Endocrinology and Metabolism, The First Affiliated Hospital of Nanjing Medical University, Jiangsu Province Hospital, Nanjing, 210029 China; 2 Department of Geriatrics, The Affiliated Hospital of Nantong University, Nantong, 226001 China; 3 Department of Endocrinology and Metabolism, The Affiliated Huai’an No.1 People’s Hospital of Nanjing Medical University, Huai’an, 223001 China; 4 Department of Endocrinology, The Second People’s Hospital of Huai’an, The Affiliated Huai’an Hospital of Xuzhou Medical University, Huai’an, 223001 China; 5 Department of Endocrinology, The Affiliated Suzhou Science & Technology Town Hospital of Nanjing Medical University, Suzhou, 215000 China; 6 Department of Endocrinology, The Affiliated Hospital of Jiangsu University, Zhenjiang, 212000 China; 7 Department of Endocrinology, The First People’s Hospital of Chuzhou, Chuzhou, 239000 China; 8 Department of Endocrinology, Northern Jiangsu People’s Hospital, Yangzhou University, Yangzhou, 225000 China; 9 Department of Endocrinology, The Second Affiliated Hospital of Dalian Medical University, Dalian, 116000 China; 10 Department of Biostatistics, School of Public Health, Nanjing Medical University, Nanjing, 210029 China; 11 Department of Endocrinology, Suzhou Municipal Hospital Affiliated to Nanjing Medical University, Suzhou, 215000 China; 12 Department of Endocrinology, The First Affiliated Hospital of Soochow University, Suzhou, 215000 China; 13 Department of Endocrinology, The Affiliated Changzhou No. 2 People’s Hospital of Nanjing Medical University, Changzhou, 213000 China

**Keywords:** Companion-intensive multi-aspect lifestyle intervention, Obesity, Weight loss, Body composition

## Abstract

**Background:**

Obesity is a globally increasing health epidemic requiring early lifestyle intervention. Our main objective was to examine the effectiveness of companion-intensive multi-aspect weight management (CIMWM) in Chinese adults with obesity.

**Methods:**

In this 6-month, prospective, open-label, multicenter, randomized controlled clinical trial, we recruited 272 obese adults aged 18–50 years with a body mass index (BMI) ≥ 28.0 kg/m^2^ and capable of using smartphones. CIMWM (n = 136) offered both daily online instructions and monthly face-to-face guidance by physicians, dietitians, and health managers along with the provision of meal replacements in the first 3 months. Traditional multi-aspect weight management (TMWM, n = 136) provided monthly face-to-face guidance by the same panel of professionals and the same meal replacements as CIMWM group, but required subjects to complete daily self-monitoring instead of offering daily online instructions. Body composition and metabolic parameters were assessed at baseline, 1, 2, 3, and 6 months by physicians. The primary outcomes were clinically-significant weight loss and changes in BMI and body composition.

**Results:**

Participants in both groups showed significantly reduced BMI, body fat mass (BFM), visceral fat area (VFA), and HOMA-IR (*p* < 0.05). CIMWM was shown to be superior to TMWM in the improvement of clinically-significant weight loss, BMI, total cholesterol (TC), the body composition parameters BFM and the skeletal muscle mass-to-visceral fat area ratio (S/V) (*p* < 0.05). The non-alcoholic fatty liver disease score (NFS) was negatively related to S/V at baseline. After weight management, NFS was lowered among individuals with levels in the highest tertile (*p* < 0.05). Metabolic memory in terms of the continuous reduction of BMI, BFM, and TC was retained up to 6 months in spite of participants transferring to self-monitoring assessment in the final 3 months.

**Conclusions:**

The CIMWM strategy in obese Chinese adults is proved to be more effective than TMWM in weight loss, and motivates greater adherence to intervention and lifestyle reprogramming.

*Trial registration* Chinese Clinical Trial Registry, ChiCTR1800017463, Registered July 31, 2018. http://www.chictr.org.cn/showproj.aspx?proj=29649.

## Background

Obesity is a global health epidemic. According to the World Health Organization (WHO), up to 57.8% of adults worldwide will be classified as obese by 2030 [1]. The prevalence of obesity among Chinese adults is ~ 11% [2]. Body mass index (BMI) is a widely used parameter to define overweight and obese status in adults [3]. In China, individuals within a BMI range of 24.0–27.9 kg/m^2^ are defined as overweight and those with BMI ≥ 28.0 kg/m^2^ as obese [4].

Obesity is an underlying risk factor for a series of disorders, such as diabetes, non-alcoholic fatty liver disease (NAFLD), coronary heart disease, sleep apnea syndrome, stroke, and cancer, which are associated with significantly increased morbidity and mortality [5, 6]. Effective treatment options and prevention strategies to control obesity are therefore a top priority for healthcare systems [7]. Scientifically sound and appropriate weight loss treatments are essential to lower the risk of obesity-related diseases. Weight loss treatments that have been proven effective for patients with obesity include lifestyle modification, conventional pharmacologic treatments and surgical treatments [8]. In particular, lifestyle modification is the foundation of weight loss and should be consistently implemented [9].

Intensive lifestyle modification involves alterations in five indispensable aspects: diet, exercise, psychotherapy, behavioral intervention, and health education. The Diabetes Remission Clinical Trial (DiRECT) in the UK showed significant benefits in patients with type 2 diabetes mellitus (T2DM) and obesity who underwent a primary care-led weight management program, with almost half of the participants achieving remission to a non-diabetic state and requiring no further treatment by 12 months [10]. Another study demonstrated that a Web-Based Behavior Change Program could help achieve weight loss and reduce cardiovascular disease (CVD) risk in obese individuals, supporting the internet as a viable medium for weight loss management [7]. Records showed that in 2014, ~ 92% of the population in China had access to mobile phones, with smartphones being widely used by young and middle-aged individuals [11].

Although early lifestyle intervention is important for the control of obesity, it is difficult for patients to adhere to daily modifications on their own, so that intensive supervision by doctors or other professionals is necessary for successful weight management. Irregular face-to-face guidance by the community and hospitals is the traditional method used for patients with obesity in China, which often results in poor achievement of self-monitoring ability and weight loss. To resolve this issue, we proposed the implementation of a Companion-Intensive Multi-aspect Weight Management (CIMWM) strategy focusing on a combination of online and offline medical interventions with daily lifestyle supervision and guidance of diet and exercise. To evaluate this strategy, we conducted a multicenter, randomized, controlled clinical intervention trial for adults with obesity in China from 2018 to 2019, which aimed to compare the efficacy and feasibility of two different medical weight management intervention programs. CIMWM offers both daily online instructions and monthly face-to-face guidance by physicians, dietitians and health managers, along with provision of meal replacements in the first 3 months. Individuals of traditional multi-aspect weight management (TMWM) were provided with monthly face-to-face guidance by the same panel of professionals and the same meal replacements as CIMWM group, but were required to complete daily self-monitoring instead of being offered with daily online instructions. Changes in clinical indicators, such as lipid profiles, blood glucose, Homeostatic Model Assessment of Insulin Resistance (HOMA-IR), blood pressure, and body composition, were additionally evaluated. This study is beneficial in raising public awareness of obesity prevention, proposing the appropriate obesity intervention programs, and providing evidence for relevant guidelines and formulation of local government health policies [12].

## Methods

### Study design

This study was designed as a prospective, open, multicenter, 6-month, randomized, controlled clinical trial, registered at the Chinese Clinical Trial Registry (ChiCTR1800017463), and approved by the Ethics Committee at the First Affiliated Hospital of Nanjing Medical University (Nanjing, Jiangsu, China) (2018-SR-069). The recruitment period was from August 1, 2018 to June 31, 2019. Recruitment strategies included advertisements in social media, e.g. WeChat, along with posters and referrals from the outpatient clinic. Written informed consents were obtained from all participants.

### Participants

This clinical trial was conducted primarily in 11 tertiary care hospitals across China, including (1) 86 patients from the First Affiliated Hospital of Nanjing Medical University (Jiangsu Province Hospital, Primary center), (2) 20 patients from the Affiliated Changzhou No. 2 People’s Hospital of Nanjing Medical University, with 4 withdrawn, (3) 28 patients from the First Affiliated Hospital of Soochow University, with 3 withdrawn, (4) 20 patients from Suzhou Municipal Hospital Affiliated to Nanjing Medical University, with 1 withdrawn, (5) 10 patients from the Affiliated Suzhou Science &Technology Town Hospital of Nanjing Medical University, with 1 withdrawn, (6) 19 patients from the Affiliated Hospital of Jiangsu University, (7) 13 patients from the Affiliated Huai’an No.1 People’s Hospital of Nanjing Medical University, (8) 27 patients from the Second People's Hospital of Huai'an, the Affiliated Huai’an Hospital of Xuzhou Medical University, (9) 15 patients from Northern Jiangsu People’s Hospital, (10) 25 patients from the First People’s Hospital of Chuzhou, with 9 withdrawn, and (11) 9 patients from the Second Affiliated Hospital of Dalian Medical University.

The duration of the study was from July 1, 2018 to December 30, 2019.

### Inclusion and exclusion criteria

**Fig. 1:**
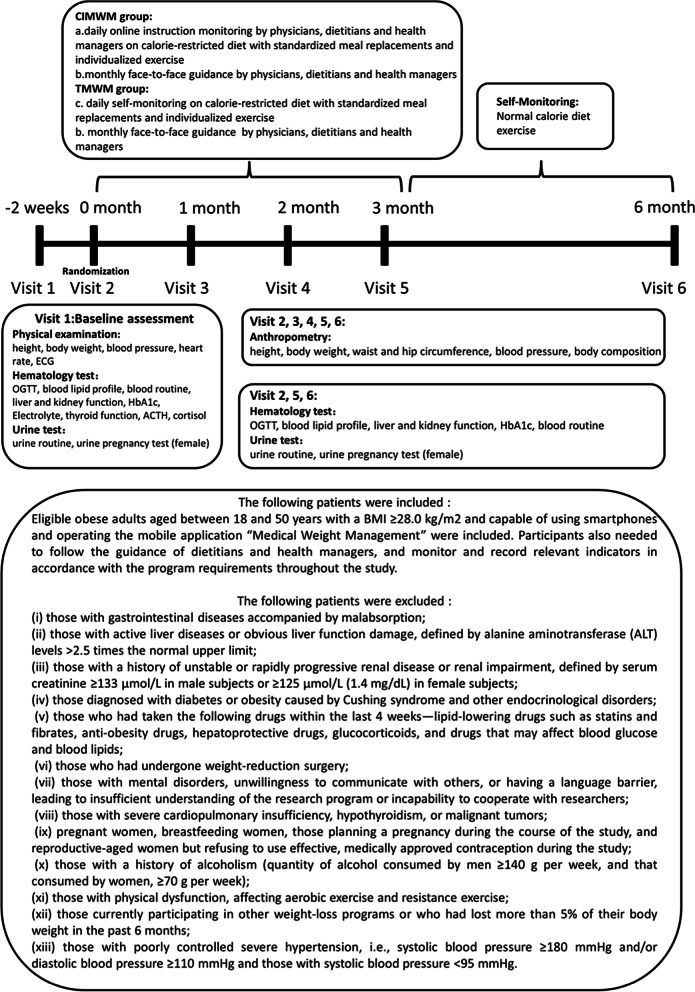
Structure of the weight management programs

Eligible adults with obesity aged between 18 and 50 years with BMI ≥ 28.0 kg/m^2^, capable of using smartphones and operating the mobile application “Medical Weight Management” (Guangzhou ND-fit Nutrition and Health Consulting Co. Ltd) were included. Participants additionally needed to follow the guidance of dietitians and health managers, and monitor relevant indicators in accordance with the program requirements throughout the study. The complete inclusion and exclusion criteria are presented in Fig. 1.

### Intervention

A multi-aspect team comprising physicians, dietitians, and health managers delivered the program over a 6-month period. Participants in the CIMWM group were provided with two Fit Nutrition Bars (Guangzhou ND-fit Nutrition and Health Consulting Co. Ltd) per day in the first 3 months as well as monthly face-to-face guidance and daily online instructions via the mobile application “Medical Weight Management”, which allowed uploading data of daily weight and food diaries. This information helped our team of experts to manage and guide the subjects online in real time. The TMWM group underwent a separate program with monthly face-to-face guidance by the same multi-aspect team and the same meal replacements, but with daily self-monitoring instead of a real-time guidance via mobile application. In the final 3 months, all participants were transferred to the self-monitoring period of the program. All participants were followed up at 1, 2, 3, and 6 months after randomization for assessment. Details of the interventions are presented in Fig. 1.

Features of the diet and exercise intervention programs are described below. Based on age, sex, standard body weight, typical caloric intake, and physical activities, participants received an individualized calorie-restricted diet (CRD) [13] plan with 1200–1800 kilocalories (kcal) per day, which was developed by registered dietitians, providing 40–55% of daily calories from carbohydrates, 20–30% from fat, and 15–20% from protein. Fit Nutrition Bars consisting of whey protein, soy protein isolate, chia seeds, oligosaccharides, collagen, and konjac extract rich in dietary fiber and γ-aminobutyric acid were provided as meal replacements for participants from both groups in the first 3 months. A single bar weighs 30 g and provides 111 kcal with 9.15 g protein, 1.8 g fat, 12.48 g carbohydrate, and 3.93 g dietary fiber. Both groups were additionally provided with calcium, vitamin D, and multivitamin mineral tablets.

Individualized exercise plans were created by health managers for each participant based on their health status and exercise capacity. The exercise plan included a weekly 160 min group exercise session. Over 6 months, participants were instructed to exercise for 40 min/day, 4 days a week, starting with 5 min warm-up exercises, followed by 10 min and 30 min of resistance and aerobic exercises, respectively, and ending with 5 min muscle stretching exercises.

### Assessments

Assessments were conducted face-to-face at baseline, 1, 2, 3, and 6 months by physicians.

#### Primary outcomes

Clinically-significant weight loss (defined as weight loss ≥ 5%) in different weight managements groups was one of the primary outcomes. Body mass index (BMI), body weight (BW), body fat percentage (BFP), body fat mass (BFM), fat-free mass (FFM), skeletal muscle mass (SMM), and visceral fat area (VFA) were measured in participants while standing on an automated hand-to-foot bioelectrical impendence device (JAWON IOI353 Body composition analyzer, Korea) with bare feet and light clothing.

#### Secondary outcomes

Anthropometric measurements, such as height, waist circumference (WC) and hip circumference (HC) accurate to the nearest 0.01 cm, were obtained using standard techniques. Blood pressure (while seated) was recorded as the average of two measures. Blood samples were obtained in the morning after an overnight fast. The BECKMAN-AU5800 automatic biochemical analyzer was used for determination of fasting plasma glucose (FPG), alanine aminotransferase (ALT), aspartate aminotransferase (AST), total cholesterol (TC), triglyceride (TG), high-density lipoprotein cholesterol (HDL-C), low-density lipoprotein cholesterol (LDL-C), lipoprotein(a) [Lp(a)], creatinine (Cr), uric acid (UA), platelet (PLT), and albumin (ALB) levels. Fasting insulin (FINS) and fasting C peptide (FCP) contents were determined via chemiluminescence, and glycated hemoglobin (HbA1c) was detected using high-performance liquid chromatography (Bole, USA). The non-alcoholic fatty liver disease score (NFS) was calculated as follows:

− 1.675 + 0.037 × age (years) + 0.094 × BMI (kg/m^2^) + 1.13 × impaired fasting glucose/diabetes (yes = 1, no = 0) + 0.99 × AST/ALT ratio − 0.013 × platelet count (× 10^9^/L) − 0.66 × albumin (g/dL) [14].

Advanced fibrosis was accurately excluded by applying the NFS low cut-off point (− 1.455) while its presence was diagnosed with high accuracy by applying the NFS high cut-off point (0.676) [14]. HOMA-IR was calculated using the formula: FPG (mmol/L) × FINS (mIU/L)/22.5.

Diabetes mellitus was defined as either FPG ≥ 7 mmol/L or 2 h oral glucose tolerance test (OGTT) blood glucose (2hBG) ≥ 11.1 mmol/L, and impaired fasting glucose (IFG) was defined as 6.1 mmol/L ≤ FPG < 7 mmol/L and 2hBG < 7.8 mmol/L[15].

### Statistical analysis

This study was powered using two previous technology-based weight management studies [16, 17], based on the percentage of individuals with clinically-significant weight loss (defined as weight loss ≥ 5%) in groups using different weight managements. A sample size of 214 subjects, 107 in each arm, is sufficient to detect a clinically important difference of about 20% between groups using a two-tailed z-test of proportions with 90% power and a 5% level of significance. Considering a dropout rate of 20%, the sample size required is 272 (136 per group). Accordingly, competitive enrollment was initiated at each branch center. Enrolled patients were numbered in adjacent sequences using the randomized block design (block size = 4). Random assignment codes were generated by statistics professionals using the SAS 9.2 software proc plan program according to a 1:1 allocation ratio [7].

Analyses were performed using SPSS for Macintosh version 25.0 (SPSS Inc, Chicago, IL, USA) and SAS 9.2 software proc plan program. Demographic and baseline characteristics were evaluated with the aid of descriptive statistics. Data are represented as mean ± SD for continuous variables and as percentages for categorical variables. Data sets involving the baseline, 1-month, 2-month, 3-month, and 6-month index of the participants in each group were assessed using repeated measurement ANOVA. Spearman’s rank correlation was performed to evaluate the relationships between the changes of physical examination and hematology indices at the 6-month point. To compare the characteristics of continuous variables between the two groups, the differences of changes from baseline to 1, 2, 3, and 6 months between two groups were assessed with mixed model controlling for baseline index using SAS 9.2. For categorical variables, the chi-square test was used. Two-sided *p* values < 0.05 were considered statistically significant.

## Results

### Participants

**Fig. 2:**
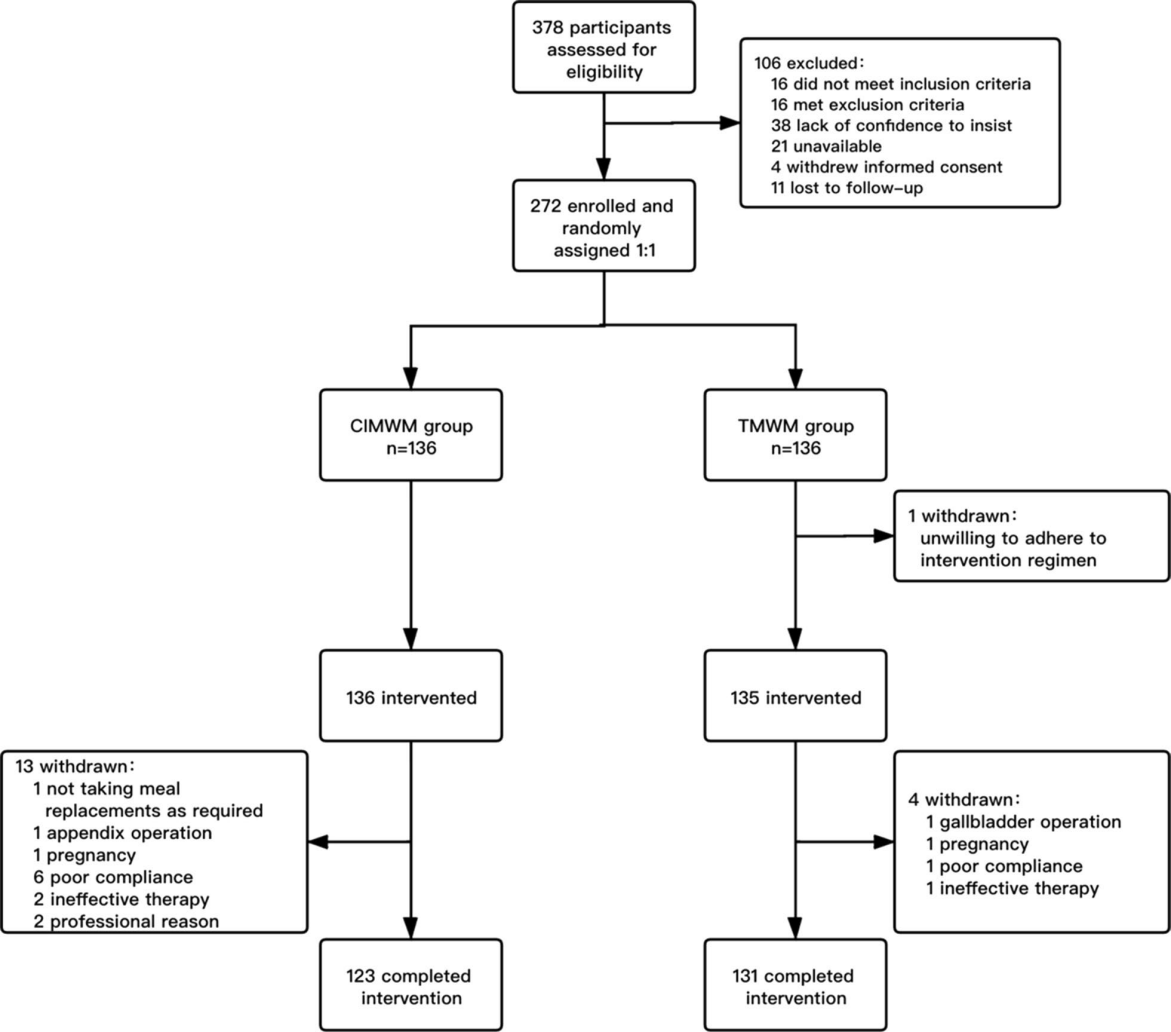
Flow chart of participants through recruitment and follow-up

Participants were recruited from August 2018 to June 2019 (Fig. 2). Among the 378 eligible subjects, 272 (72%) were included and randomly assigned to study groups at a 1:1 allocation ratio. Overall, 254 (93.3%) participants completed the 6-month weight management intervention study.

### Anthropometric, metabolic, and clinical characteristics of the participants in each group before and after the weight loss intervention

The baseline characteristics of both groups are presented in Table 1. No significant intergroup differences were observed except for FCP.

**Table 1:** Baseline characteristics of participants in the CIMWM and TMWM groups^a^

	CIMWM	TMWM	*p* value^b^
Age (years)	31.52 ± 6.43	32.16 ± 6.39	0.427
Sex (M/F)	72/51	77/54	0.969
BMI (kg/m^2^)	32.36 ± 3.40	32.48 ± 3.59	0.800
BW (kg)	92.77 ± 14.91	92.98 ± 14.08	0.909
BFP (%)	34.60 ± 4.54	35.03 ± 4.72	0.459
BFM (kg)	32.03 ± 6.32	32.61 ± 7.06	0.490
FFM (kg)	60.82 ± 11.14	60.30 ± 9.88	0.689
SMM (kg)	33.30 ± 6.20	33.07 ± 5.49	0.752
VFA (cm^2^)	133.70 ± 34.46	140.5 ± 48.59	0.205
S/V (kg/cm^2^)	0.26 ± 0.08	0.27 ± 0.23	0.760
WC (cm)	103.59 ± 11.78	103.70 ± 12.74	0.940
HC (cm)	110.61 ± 7.58	110.34 ± 11.86	0.832
WHR	0.94 ± 0.09	0.99 ± 0.69	0.365
SBP (mmHg)	130.88 ± 12.79	131.73 ± 13.64	0.607
DBP (mmHg)	82.66 ± 10.22	83.75 ± 10.70	0.409
ALT (U/L)	44.79 ± 37.24	38.36 ± 26.57	0.113
AST (U/L)	27.98 ± 14.08	26.02 ± 11.46	0.224
TC (mmol/L)	5.15 ± 1.05	5.09 ± 0.84	0.574
TG (mmol/L)	2.16 ± 1.70	1.98 ± 1.12	0.307
HDL-C (mmol/L)	1.11 ± 0.27	1.22 ± 1.16	0.296
LDL-C (mmol/L)	3.19 ± 0.74	3.17 ± 0.71	0.839
Lp (a) (mg/L)	158.20 ± 172.72	140.05 ± 145.57	0.365
FPG (mmol/L)	5.16 ± 0.75	5.13 ± 1.08	0.763
Cr (µmol/L)	70.25 ± 15.70	67.57 ± 15.71	0.176
UA (µmol/L)	404.12 ± 111.32	389.11 ± 116.72	0.296
FINS (pmol/L)	157.82 ± 198.46	135.32 ± 80.83	0.233
FCP (pmol/L)	1292.20 ± 1201.69	1062.05 ± 454.12	0.048*
HbA1c (%)	5.51 ± 0.42	5.48 ± 0.37	0.516
IFG Number (%)	11(8.9%)	10(7.6%)	0.705
PLT (×10^9^)	267.80 ± 56.57	261.10 ± 60.25	0.363
ALB (g/L)	45.90 ± 4.26	45.48 ± 3.96	0.418
NFS	− 3.12 ± 1.01	− 2.91 ± 1.07	0.111
Fibrosis Severity Scale			0.126
F0–F2	119(96.7%)	121(92.4%)	
Indeterminant score	4(3.3%)	10(7.6%)	
F3–F4	0	0	
HOMA-IR	5.16 ± 6.15	4.42 ± 2.78	0.209

*BMI* Body mass index, *BW* body weight, *BFP* body fat percentage, *BFM* body fat mass, *FFM* fat-free mass, *SMM* skeletal muscle mass, *VFA* visceral fat area, *S/V* skeletal muscle mass-to-visceral fat area radio, *WC* waist circumference, *HC* hip circumference, *WHR* waist-to-hip ratio, *ALT* alanine aminotransferase, *AST* aspartate aminotransferase, *TC* total cholesterol, *TG* triglyceride, *HDL⁃C* high-density lipoprotein cholesterol, *LDL*-*C* low-density lipoprotein cholesterol, [*Lp(a)*] Lipoprotein(a), *Cr* creatinine, *UA* uric acid, *FPG* fasting plasma glucose, *FINS* fasting insulin, *FCP* fasting C peptide, *PLT* platelet, *ALB* albumin, *HbA1c* glycated hemoglobin, *NFS* non-alcoholic fatty liver disease score, *IFG* impaired fasting glucose, *SBP* systolic blood pressure, *DBP* diastolic blood pressure, *HOMA-IR* homeostasis model assessment of insulin resistance

^a^Data are represented as mean ± SD for continuous variables and as percentages for categorical variables

^b^Independent samples *t* test and chi-square test were used for continuous and categorical variables, respectively

*p < 0.05

**Fig. 3:**
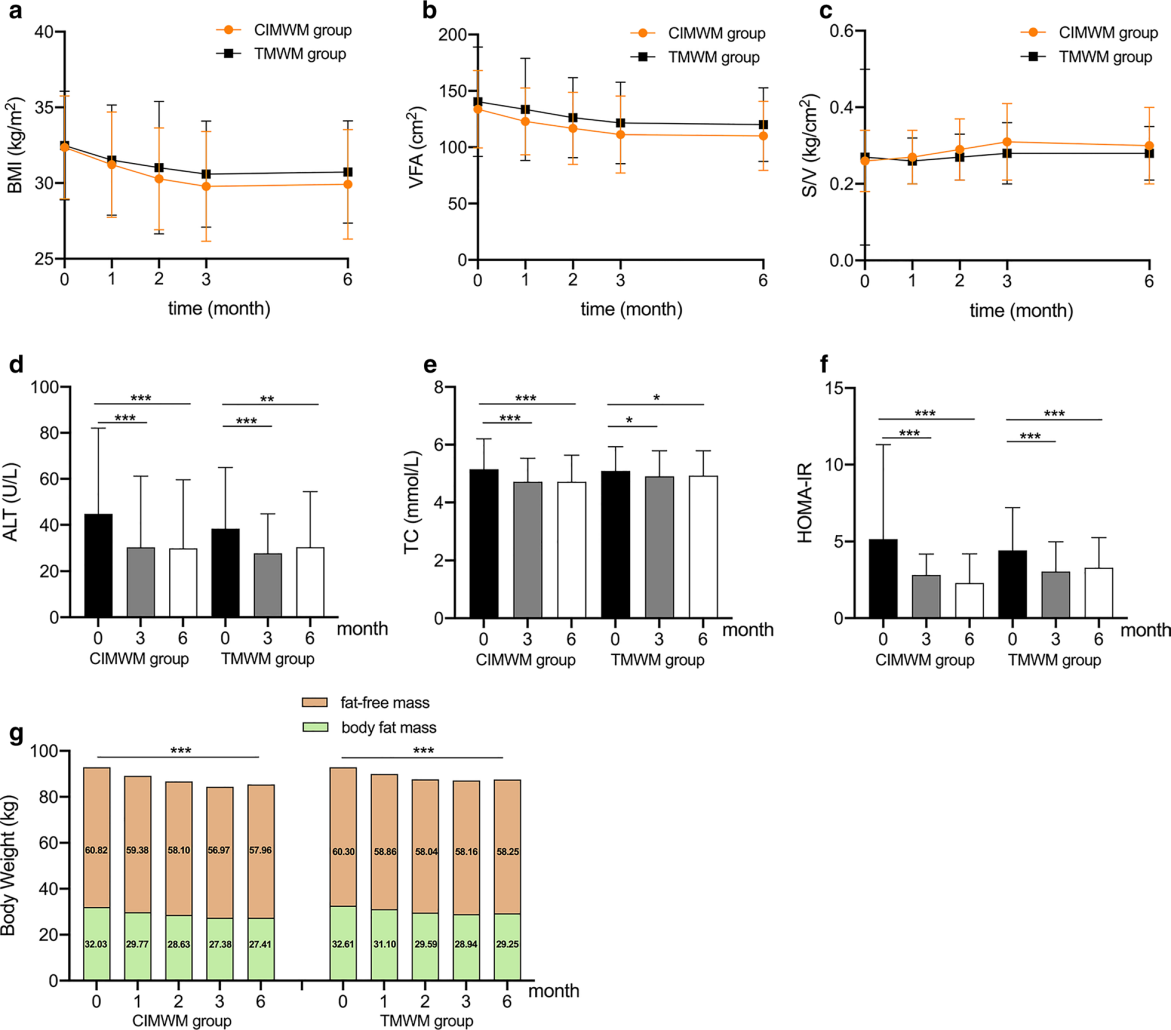
BMI, VFA, S/V, ALT, TC, HOMA-IR, BFM and FFM changes in CIMWM and TMWM groups. *p < 0.05, **p < 0.01, ***p < 0.001, body mass index (BMI), visceral fat area (VFA), skeletal muscle mass-to-visceral fat area radio (S/V), alanine aminotransferase (ALT), total cholesterol (TC), homeostasis model assessment of insulin resistance (HOMA-IR), body fat mass (BFM), fat-free mass (FFM), Companion-Intensive Multi-aspect Weight Management (CIMWM), Traditional Multi-aspect Weight Management (TMWM). **a** Changes in BMI from baseline to 6 months respectively in CIMWM and TMWM. **b** Changes in VFA from baseline to 6 months respectively in CIMWM and TMWM. **c** Changes in S/V from baseline to 6 months respectively in CIMWM and TMWM. **d** Changes in ALT from baseline to 6 months respectively in CIMWM and TMWM. **e** Changes in TC from baseline to 6 months respectively in CIMWM and TMWM. **f** Changes in HOMA-IR from baseline to 6 months respectively in CIMWM and TMWM. **g** BFM and FFM reduction from baseline to 6 months respectively in CIMWM and TMWM

The clinical and demographic characteristics of the participants in each group are presented in Additional file 1: Table S1. Compared to baseline, BMI in both groups was significantly decreased at each visit point (*p* < 0.001, Fig. 3a). Loss of body weight, including BFM and SMM was sustained during the whole 6 months in both groups when comparing with baseline (*p* < 0.001, Additional file 1: Table S1, Fig. 3g). Additionally, BFP, BFM, VFA, WC, HC, SBP, DBP, ALT, TC, TG, LDL-C, FINS, and HOMA-IR, were significantly decreased at both 3 and 6 months (*p* < 0.05, Additional file 1^1^ Table S1, Fig. 3b, d, e, f). Nevertheless, at 6 months compared with baseline, FFM was slightly decreased by 2.86 kg in CIMWM group (*p* < 0.001) and 2.05 kg in TMWM group (*p* < 0.001), while SMM was decreased by 2.06 kg in CIMWM group (*p* < 0.001) and 1.48 kg in TMWM group (*p* < 0.001). However, sustaining increase of S/V over the 6 months was only observed in the CIMWM group, compared with baseline (*p* < 0.001, Fig. 3c). Additionally, only in the CIMWM group, the number of subjects with IFG was significantly decreased at both 3 and 6 months. Moreover, only at 6 months, UA was markedly declined from baseline in both groups. HDL-C levels was gradually increased from baseline to the end of the study period in CIMWM group (*p* < 0.001).

**Fig. 4:**
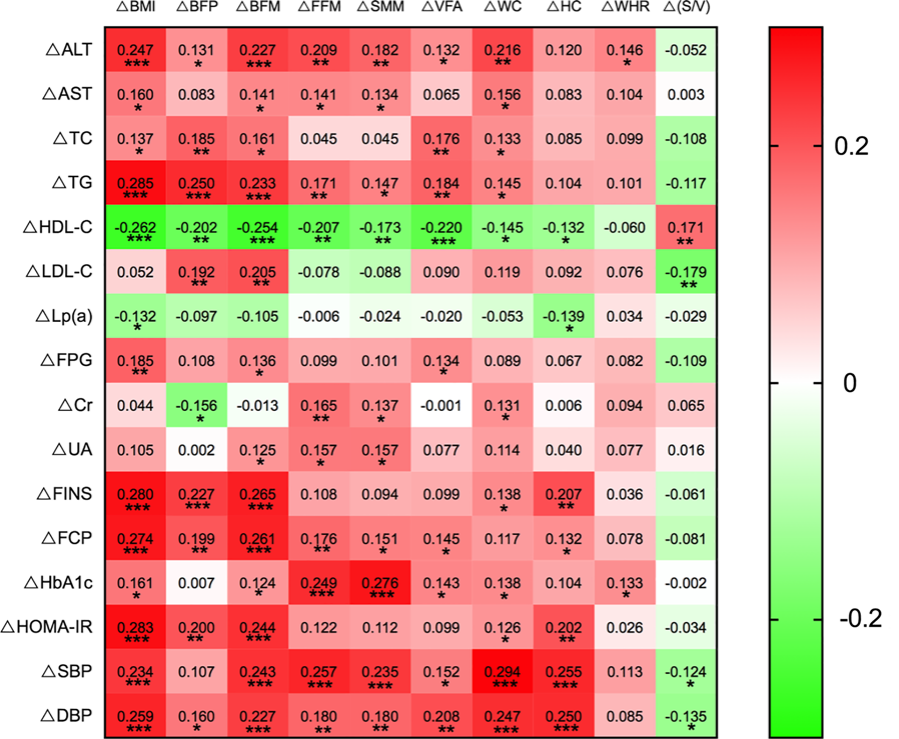
Spearman’s correlation coefficient heatmap among physical and hematological indices changes from baseline to 6 months. Body mass index (BMI), body fat percentage (BFP), body fat mass (BFM), fat-free mass (FFM), skeletal muscle mass (SMM), visceral fat area (VFA), skeletal muscle mass-to-visceral fat area radio (S/V), waist circumference (WC), hip circumference (HC), waist-to-hip ratio (WHR), alanine aminotransferase (ALT), aspartate aminotransferase (AST), total cholesterol (TC), triglyceride (TG), high-density lipoprotein cholesterol (HDL⁃C), low-density lipoprotein cholesterol (LDL-C), lipoprotein(a) [Lp(a)], creatinine (Cr), uric acid (UA), fasting plasma glucose (FPG), fasting insulin (FINS), fasting C peptide (FCP), glycated hemoglobin (HbA1c), systolic blood pressure (SBP), diastolic blood pressure (DBP), homeostasis model assessment of insulin resistance (HOMA-IR). *p < 0.05, **p < 0.01; ***p < 0.001

The correlation coefficient heatmap (Fig. 4) indicated that the variations in BMI and BFM from baseline to 6 months were positively correlated with those of ALT, AST, TC, TG, FPG, FINS, FCP, HbA1c, HOMA-IR, SBP, and DBP (all *p* < 0.05) and inversely correlated with HDL-C changes (*p* < 0.05). Positive associations were observed between 6-month changes of VFA and ALT, TC, TG, FPG, FCP, HbA1c, SBP, as well as DBP values (*p* < 0.05). Simultaneously, changes in S/V were positively correlated with HDL-C and negatively correlated with LDL-C, SBP, and DBP.

### NFS values in tertile subgroups and relationships between baseline NFS and anthropometrical parameters

**Fig. 5:**
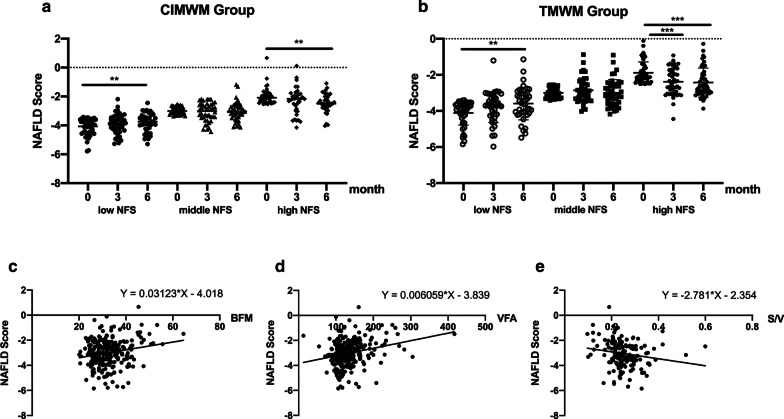
NFS changes in tertile subgroups and relationships between baseline NFS and anthropometrical parameters. **p < 0.01; ***p < 0.001, Non-alcoholic fatty liver disease (NAFLD) score (NFS), body fat mass (BFM), visceral fat area (VFA), Skeletal muscle mass-to-visceral fat area radio(S/V). **a** Comparison of NFS from subgroups in CIMWM based on tertiles at baseline. **b** Comparison of NFS from subgroups in TMWM based on tertiles at baseline. **c** Relationship between baseline NFS and BFM. **d** Relationship between baseline NFS and VFA. **e** Relationship between baseline NFS and S/V

Baseline NFS was positively associated with BFM and VFA and negatively correlated with S/V (*p* < 0.05; Fig. 5 c, d, e). No difference in NFS was observed at 0, 3, and 6 months in both groups. To further validate the changes in NFS, 123 participants in CIMWM group and 131 participants in TMWM group were respectively stratified into three groups based on the tertiles (− 3.41 and − 2.53) of NFS at baseline. NFS of the highest tertile group gradually decreased over the subsequent 6 months to a significantly different extent from baseline (*p* < 0.05). However, in the lowest tertile, a significant increase was observed at 6 months, which only happened in several participants, while, whose values still remained below the high cut-off point (0.676) of liver fibrosis (Fig. 5a, b).

### Outcome differences between CIMWM and TMWM groups during the 6-month follow-up

From 2 months, the difference started to manifest significance between individuals achieving clinically-significant weight loss (defined as weight loss ≥ 5%) from two groups (*p* = 0.003, Table 2). At 3 months, there were more obvious differences in achievement of clinically-significant weight loss between groups (*p* < 0.001). At 6 months, the significant difference between the CIMWM (66.7%) and TMWM (54.2%) groups was still maintained (*p* = 0.042).

**Table 2:** Outcome differences between CIMWM and TMWM groups during the 6-month follow-up period^a^

	Time (month)	CIMWM	TMWM	*p* value^b^
Weight loss ≥ 5%	1	30.9% (38/123)	24.4% (32/131)	0.249
	2	68.3% (84/123)	49.6% (65/131)	0.003**
	3	76.4% (94/123)	54.2% (71/131)	< 0.001***
	6	66.7% (82/123)	54.2% (71/131)	0.042*
△BMI (kg/m^2^)	1	− 1.14 ± 1.17	− 0.96 ± 1.05	0.437
	2	− 2.08 ± 1.21	− 1.46 ± 3.84	0.013*
	3	− 2.58 ± 1.51	− 1.88 ± 2.12	0.006**
	6	− 2.44 ± 1.82	− 1.74 ± 2.59	0.006**
△BW (kg)	1	− 3.65 ± 2.31	− 3.06 ± 3.70	0.438
	2	− 6.30 ± 5.62	− 5.42 ± 7.55	0.260
	3	− 8.09 ± 6.57	− 5.77 ± 7.90	0.004**
	6	− 7.42 ± 8.16	− 6.86 ± 10.67	0.461
△BFP (%)	1	− 1.13 ± 1.50	− 0.60 ± 1.45	0.062
	2	− 1.82 ± 2.14	− 1.33 ± 2.46	0.085
	3	− 2.43 ± 2.77	− 1.89 ± 2.52	0.058
	6	− 2.74 ± 3.26	− 1.94 ± 3.02	0.006**
△BFM (kg)	1	− 2.26 ± 1.79	− 1.51 ± 1.72	0.055
	2	− 3.40 ± 3.13	− 3.02 ± 4.20	0.284
	3	− 4.64 ± 2.80	− 3.67 ± 4.21	0.015*
	6	− 4.62 ± 3.54	− 3.36 ± 5.67	0.002**
△FFM (kg)	1	− 1.44 ± 1.86	− 1.43 ± 3.15	0.873
	2	− 2.73 ± 4.73	− 2.25 ± 4.99	0.498
	3	− 3.86 ± 6.52	− 2.14 ± 5.22	0.004**
	6	− 2.86 ± 5.59	− 2.05 ± 5.15	0.203
△SMM (kg)	1	− 0.78 ± 1.41	− 0.80 ± 1.72	0.871
	2	− 1.29 ± 2.40	− 1.21 ± 2.68	0.925
	3	− 1.08 ± 4.47	− 1.11 ± 2.60	0.855
	6	− 2.06 ± 4.71	− 1.48 ± 3.63	0.149
△VFA (cm^2^)	1	− 10.86 ± 19.05	− 6.84 ± 25.88	0.028*
	2	− 17.00 ± 21.87	− 14.13 ± 36.58	0.067
	3	− 22.38 ± 23.52	− 18.90 ± 36.28	0.043*
	6	− 23.70 ± 28.21	− 20.45 ± 39.15	0.051
△(S/V) (kg/cm^2^)	1	0.01 ± 0.05	− 0.01 ± 0.22	0.061
	2	0.03 ± 0.06	− 0.00 ± 0.22	0.012*
	3	0.05 ± 0.07	0.01 ± 0.23	0.002**
	6	0.04 ± 0.08	0.01 ± 0.22	0.008**
△WC (cm)	1	− 2.29 ± 7.22	− 1.35 ± 9.95	0.322
	2	− 6.14 ± 11.56	− 4.19 ± 10.39	0.047*
	3	− 7.12 ± 7.80	− 5.35 ± 11.78	0.071
	6	− 7.33 ± 8.93	− 5.70 ± 12.25	0.095
△HC (cm)	1	− 2.13 ± 4.46	− 0.76 ± 9.84	0.218
	2	− 4.34 ± 5.45	− 4.40 ± 14.24	0.833
	3	− 5.71 ± 5.81	− 4.39 ± 13.94	0.241
	6	− 4.95 ± 5.48	− 3.83 ± 11.09	0.329
△WHR	1	− 0.00 ± 0.07	− 0.06 ± 0.69	0.931
	2	− 0.02 ± 0.10	− 0.04 ± 0.72	0.936
	3	− 0.02 ± 0.08	− 0.05 ± 0.72	0.164
	6	− 0.03 ± 0.08	− 0.07 ± 0.70	0.936
△SBP (mmHg)	1	− 4.92 ± 11.27	− 4.49 ± 12.44	0.483
	2	− 6.38 ± 12.33	− 4.91 ± 13.47	0.136
	3	− 7.11 ± 13.17	− 7.21 ± 12.97	0.772
	6	− 6.66 ± 12.97	− 6.32 ± 13.64	0.531
△DBP (mmHg)	1	− 4.62 ± 10.17	− 4.33 ± 10.31	0.305
	2	− 5.94 ± 9.98	− 4.34 ± 9.83	0.017*
	3	− 6.24 ± 10.19	− 5.73 ± 10.53	0.208
	6	− 5.64 ± 10.25	− 6.14 ± 10.52	0.829
△ALT (U/L)	3	− 14.40 ± 21.74	− 10.61 ± 24.03	0.836
	6	− 14.86 ± 25.98	− 7.94 ± 31.41	0.156
△AST (U/L)	3	− 6.32 ± 10.49	− 4.50 ± 10.08	0.548
	6	− 5.62 ± 12.86	− 2.48 ± 14.37	0.095
△TC (mmol/L)	3	− 0.43 ± 0.94	− 0.18 ± 0.85	0.025*
	6	− 0.43 ± 0.97	− 0.16 ± 0.85	0.013*
△TG (mmol/L)	3	− 0.76 ± 1.79	− 0.60 ± 0.96	0.849
	6	− 0.48 ± 1.63	− 0.30 ± 1.17	0.713
△HDL-C (mmol/L)	3	0.03 ± 0.23	0.02 ± 1.42	0.339
	6	0.10 ± 0.27	0.02 ± 1.15	0.283
△LDL-C (mmol/L)	3	− 0.17 ± 0.65	− 0.06 ± 0.72	0.168
	6	− 0.27 ± 0.67	− 0.15 ± 0.72	0.126
△Lp (a) (mg/L)	3	42.88 ± 149.61	49.15 ± 86.74	0.881
	6	47.91 ± 193.69	25.69 ± 94.77	0.122
△FPG (mmol/L)	3	− 0.26 ± 0.74	− 0.19 ± 1.06	0.674
	6	− 0.23 ± 0.91	− 0.18 ± 1.14	0.756
△Cr (µmol/L)	3	− 1.30 ± 9.39	0.70 ± 11.46	0.448
	6	− 0.61 ± 11.08	− 0.97 ± 14.54	0.282
△UA (µmol/L)	3	5.25 ± 71.00	− 3.48 ± 102.55	0.097
	6	− 34.21 ± 82.14	− 19.11 ± 95.79	0.361
△FINS (pmol/L)	3	− 67.95 ± 192.05	− 39.86 ± 76.70	0.229
	6	− 62.95 ± 194.36	− 32.24 ± 73.93	0.107
△FCP (pmol/L)	3	− 385.4 ± 1244.23	− 120.41 ± 698.46	0.382
	6	− 421.05 ± 1219.72	− 197.40 ± 450.50	0.872
△HbA1c (%)	3	− 0.17 ± 0.31	− 0.09 ± 0.36	0.117
	6	− 0.04 ± 0.41	− 0.02 ± 0.45	0.885
△HOMA-IR	3	− 2.35 ± 6.01	− 1.37 ± 2.76	0.182
	6	− 2.17 ± 6.08	− 1.12 ± 2.74	0.103
△NFS	3	0.01 ± 0.71	− 0.10 ± 0.86	0.749
	6	− 0.02 ± 0.74	− 0.03 ± 1.02	0.649

*BMI* body mass index, *BW* body weight, *BFP* body fat percentage, *BFM* body fat mass, *FFM* fat-free mass, *SMM* skeletal muscle mass, *VFA* visceral fat area, *S/V* skeletal muscle mass-to-visceral fat area radio, *WC* waist circumference, *HC* hip circumference, *WHR* waist-to-hip ratio, *ALT* alanine aminotransferase,* AST* aspartate aminotransferase, *TC* total cholesterol,* TG* triglyceride, *HDL-C* high-density lipoprotein cholesterol, *LDL-C* low-density lipoprotein cholesterol, [*Lp(a)*]Lipoprotein(a), *Cr* creatinine, *UA* uric acid, *FPG* fasting plasma glucose, *FINS* fasting insulin, *FCP* fasting C peptide, *HbA1c* glycated hemoglobin, *NFS* non-alcoholic fatty liver disease score, *SBP* systolic blood pressure, *DBP* diastolic blood pressure, *HOMA-IR* homeostasis model assessment of insulin resistance

^a^Data are represented as mean ± SD for continuous variables and as percentages for categorical variables

^b^Between-group differences were analyzed using mixed model controlling for baseline index; categorical variables of individuals with weight loss ≥ 5% were analyzed using chi-square analysis

*p < 0.05, **p < 0.01, ***p < 0.001

On the other hand, significant changes of BMI, BFP, BFM, S/V and TC from baseline to 6 months were observed between two groups (*p* < 0.05). Our data showed markedly lower BMI, BFM, and TC levels of the CIMWM group compared to the TMWM group during the whole study in spite of all participants transferring to self-monitoring assessment in the final 3 months (*p* < 0.05). S/V was significantly increased from baseline to 3 months in CIMWM group, and maintained during the following 3 months. Increment of S/V in the CIMWM group was significantly better than that in the TMWM group from 2 to 6 months (*p* < 0.05).

Subjects in the CIMWM group lost more body weight than those in the TMWM group at 3 months (− 8.09 kg vs. − 5.77 kg; *p* = 0.004), but this trend disappeared at 6 months (− 7.42 kg vs. − 6.86 kg; *p* = 0.461). However, there was still a marked difference in achievement of clinically significant weight loss between the CIMWM and TMWM groups at 6 months (*p* = 0.042). BMI in the CIMWM group decreased significantly by 2.08 kg/m^2^ at 2 months, 2.58 kg/m^2^ at 3 months, and 2.44 kg/m^2^ at 6 months, compared to the TMWM group (2 months: 1.46 kg/m^2^, 3 months: 1.88 kg/m^2^, 6 months, 1.74 kg/m^2^; *p* < 0.05, Table 2). We also found greater reduction of VFA in the CIMWM group at 3 months compared to the TMWM group, which was not maintained until the end of our research.

## Discussion

Results from this 6-month, randomized, controlled trial on Chinese adults with obesity showed that weight management with regular guidance by physicians, dietitians, and health managers is feasible and effective for improvement of body weight, BMI, body composition parameters (BFM and VFA), liver function, blood lipid profiles, and insulin resistance in all participants. The CIMWM strategy involving both daily online instructions and monthly face-to-face guidance was superior to TMWM with respect to improvement of BMI, TC, and body composition parameters, such as BFM, VFA, and S/V. Individuals subjected to the CIMWM program showed better adherence to guidelines and reprogramming of lifestyle. Efforts to estimate body composition changes during interventions are important in improving our awareness of metabolic health. Metabolic memory in terms of continuous reduction of BMI, BFM, and TC was retained up to 6 months, even with transfer of participants in the CIMWM group to the self-monitoring phase in the last 3 months. NFS was further evaluated to determine the degree of liver fibrosis. Our data suggest that weight management program is more beneficial in decreasing NFS among subjects with basal NFS levels in the highest tertile.

The primary outcomes of this study were the clinically-significant weight loss and reduction of BMI, BFM, and VFA from baseline to 6-month follow-up. The CIMWM group achieved significantly better reduction of these parameters than the TMWM group throughout the study. Notably, body weight increased slightly at 6 months, compared to 3 months, in both groups, but remained lower than that at baseline. We believe that the weight gain in the CIMWM group during the last 3 months of the study is attributable to the transfer to self-monitoring. Another potential reason suggested by Burke et al. [18] is a gradual decline in adherence to self-monitoring weight management program, which is common during the follow-up period. But even in the last 3 months of self-monitoring period, CIMWM group still achieved significantly better weight loss than TMWM group, suggesting the previous intense online intervention paved the way for a sustainable and positive dietary, exercise and lifestyle in their subsequent lives.

The change in body composition after weight management is an interesting phenomenon. Total body fat and distribution, especially visceral fat, is known to be associated with higher risk of metabolic syndrome [19]. Here, our data showed a reduction of BFP, BFM, and VFA in all participants, along with FFM and SMM showing a mild decrease despite physical activity support. FFM reduction is clearly associated with poor metabolic state [20–22]. Meanwhile, VFA increase and SMM decrease appear closely related to aggravation of insulin resistance and progression of NAFLD [23]. Therefore, another recommended index is the SMM to VFA radio (S/V), which simultaneously describes variations in both parameters [23]. A significant increase in S/V in all subjects was observed during the whole intervention process. Moreover, from 2 to 6 months, the increase of S/V in CIMWM group was significantly higher than that in TMWM group, suggesting the better effects of CIMWM than TMWM in the improvement of glucose metabolism and liver fat metabolism.

NFS is a clinical indicator of liver fibrosis [24]. Individuals with basal NFS  in the highest tertile displayed  significant reduction in NFS vaules after 6 months of weight intervention, indicating that our medical weight management strategy is effective not only for weight loss but also for the improvement of NAFLD. NFS in subjects starting in the lowest tertile was slightly increased, but remained under the 0.676 cut-off point for liver fibrosis. This finding is possibly related to the rebound of BMI among those individuals. However, plasma ALT and AST concentrations are still regarded as better clinical indicators than other noninvasive biomarkers/scores for definitive diagnosis of nonalcoholic steatohepatitis (NASH) and/or advanced fibrosis in patients with T2DM [25]. And we observed a significant reduction in ALT and AST levels in all participants after the intervention, which together with the NFS, ensured a well enough liver condition in this study.

At baseline, a negative correlation was observed between NFS and S/V. S/V is reported to be an independent factor that affects the controlled attenuation parameter (CAP) reflecting hepatic fat accumulation and liver stiffness measurements (LSM), representing hepatic inflammation and fibrosis [23]. In another study on NAFLD patients [24], low S/V indicated poorer hepatic condition, confirmed with liver transient elastography. Accumulating basic and clinical studies have focused on cross-talk among muscle, fat, and liver components [26]. A recent review suggests that adipose tissue function is a critical driver of NAFLD and NASH. Cytokines and hormones secreted by adipose tissue could affect the liver by regulating the lipid flux and affecting hepatocyte function via exosomal signaling [27]. Skeletal muscle is also regarded as an endocrine organ [28] that regulates myokine secretion. Circulating myonectin linking skeletal muscle to lipid metabolism in the liver and adipose tissue provides insights into tissue cross-talk, which underlies the integrated control of whole-body metabolism [29]. Hepatoprotective IL-6 [30] and irisin [31] are further reported to improve hepatic steatosis [32]. Above all, cross-talk among muscle, fat and liver might be an interesting direction for our future research on obesity.

Our study has some limitations that should be taken into consideration. Evaluation of liver steatosis and fibrosis with FibroTouch, FibroScan, or magnetic resonance elastography may present more efficient and precise results, which we didn't get a chance to obtain. In addition, we were not able to develop an accurate method to collect data on frequency and intensity of physical activities from our subjects, which may give more information on the exercise aspect for intensive lifestyle modification.

## Conclusion

Our study on weight management in Chinese adults with obesity showed that the CIMWM approach is more effective than the TMWM approach, leading to greater improvement of clinically-significant weight loss, BMI, BFM, VFA, S/V and TC parameters. The CIMWM strategy was particularly effective in motivating patient adherence to the intervention and lifestyle reprogramming. Metabolic memory in terms of continuous reduction of BMI, BFM, and TC was retained up to 6 months despite participants being transferred to the self-monitoring phase during the last 3 months of the study. Our results support the potential of a face-to-face program coupled with an online intervention tool in achieving scientifically effective management of metabolic syndrome, and providing a positive contribution to national health.

## Supplementary information

**Additional file 1: Table S1.** Anthropometric, metabolic, and clinical characteristics of participants in each group before and after the weight loss intervention.

## Data Availability

The datasets used and/or analyzed during the current study are available from the corresponding author on reasonable request.

## Abbreviations

BMI: Body mass index
BW: Body weight
BFP: Body fat percentage
BFM: Body fat mass
FFM: Fat-free mass
SMM: Skeletal muscle mass
VFA: Visceral fat area
S/V: Skeletal muscle mass-to-visceral fat area radio
WC: Waist circumference
HC: Hip circumference
WHR: Waist-to-hip ratio
SBP: Systolic blood pressure
DBP: Diastolic blood pressure
FPG: Fasting plasma glucose
ALT: Alanine aminotransferase
AST: Aspartate aminotransferase
TC: Total cholesterol
TG: Triglyceride
HDL-C: High-density lipoprotein cholesterol
LDL-C: Low-density lipoprotein cholesterol
Lp(a): Lipoprotein(a)
Cr: Creatinine
UA: Uric acid
PLT: Platelet
ALB: Albumin
FINS: Fasting insulin
FCP: Fasting C peptide
HbA1c: Glycated hemoglobin
NFS: Non-alcoholic fatty liver disease score
HOMA-IR: Homeostasis model assessment of insulin resistance
IFG: Impaired fasting glucose
