# Correction to: Effectiveness of companion-intensive multi-aspect weight management in Chinese adults with obesity: a 6-month multicenter randomized clinical trial

**DOI:** 10.1186/s12986-021-00559-y

**Published:** 2021-03-30

**Authors:** Wanzi Jiang, Shushu Huang, Shuai Ma, Yingyun Gong, Zhenzhen Fu, Li Zhou, Wen Hu, Guofang Mao, Zhimin Ma, Ling Yang, Guangfeng Tang, Xiaofang Sun, Ping Zhang, Jianling Bai, Lei Chen, Bimin Shi, Xinhua Ye, Hongwen Zhou

**Affiliations:** 1 Department of Endocrinology and Metabolism, The First Affiliated Hospital of Nanjing Medical University, Jiangsu Province Hospital, 300 Guangzhou Road, Nanjing, 210029 China; 2 Department of Geriatrics, The Affiliated Hospital of Nantong University, Nantong, 226001 China; 3 Department of Endocrinology and Metabolism, The Affiliated Huai’an No.1 People’s Hospital of Nanjing Medical University, Huai’an, 223001 China; 4 Department of Endocrinology, The Second People’s Hospital of Huai’an, The Affiliated Huai’an Hospital of Xuzhou Medical University, Huai’an, 223001 China; 5 Department of Endocrinology, The Affiliated Suzhou Science and Technology, Town Hospital of Nanjing Medical University, Suzhou, 215000 China; 6 Department of Endocrinology, The Affiliated Hospital of Jiangsu University, Zhenjiang, 212000 China; 7 Department of Endocrinology, The First People’s Hospital of Chuzhou, Chuzhou, 239000 China; 8 Department of Endocrinology, Northern Jiangsu People’s Hospital, Yangzhou University, Yangzhou, 225000 China; 9 Department of Endocrinology, The Second Affiliated Hospital of Dalian Medical University, Dalian, 116000 China; 10 Department of Biostatistics, School of Public Health, Nanjing Medical University, Nanjing, Jiangsu, 210029 China; 11 Department of Endocrinology, Suzhou Municipal Hospital Affiliated to Nanjing Medical University, Suzhou, 215000 China; 12 Department of Endocrinology, The First Affiliated Hospital of Soochow University, Suzhou, 215000 China; 13 Department of Endocrinology, The Affiliated Changzhou No. 2 People’s Hospital of Nanjing Medical University, Changzhou, 213000 China

## Correction to: Jiang et al. Nutr Metab (Lond) (2021) 18:17 https://doi.org/10.1186/s12986-020-00511-6

Following the publication of the original article [1], the authors identified that the symbol of equal contribution is missing for the authors Wanzi Jiang, Shushu Huang, Shuai Ma, and Lei Chen.

The symbol of equal contribution has been added behind the names of the authors.

Wanzi Jiang^†^, Shushu Huang^†^, Shuai Ma^†^, and Lei Chen^†^.

The author group has been updated above and the original article [1] has been corrected.
