# Supplementary material for: Effectiveness of companion-intensive multi-aspect weight management in Chinese adults with obesity: a 6-month multicenter randomized clinical trial
Source: Nutr Metab (Lond). 2021 Feb 3;18:17. doi: 10.1186/s12986-020-00511-6 (PMC7856778; doi:10.1186/s12986-020-00511-6)
Supplement: Supplementary file 1 — Additional file 1: Table S1. Anthropometric, metabolic, and clinical characteristics of participants in each group before and after the weight loss intervention. [file 12986_2020_511_MOESM1_ESM.docx]

Table S1. Anthropometric, metabolic, and clinical characteristics of participants in each group before and after the weight loss intervention ^a,b,c^

|  | Group | Baseline | 1 month | 2 months | 3 months | 6 months | P value |
| --- | --- | --- | --- | --- | --- | --- | --- |
| BMI (kg/m^2^) | CIMWM | 32.36±3.40 | 31.22±3.48^***^ | 30.28±3.37^***###^ | 29.78±3.63^***###$$$^ | 29.92±3.62^***###$$^ | <0.001 |
|  | TMWM | 32.48±3.59 | 31.52±3.64^***^ | 31.02±4.38^***^ | 30.59±3.50^***###^ | 30.73±3.37^***###^ | <0.001 |
| BW (kg) | CIMWM | 92.77±14.91 | 89.12±14.63^***^ | 86.48±13.84^***###^ | 84.68±13.65^***###$$$^ | 85.36±14.02^***###$^ | <0.001 |
|  | TMWM | 92.98±14.08 | 89.92±13.62^***^ | 87.56±12.52^***###^ | 87.21±12.03^***###^ | 86.12±12.30^***###^ | <0.001 |
| BFP (%) | CIMWM | 34.60±4.54 | 33.47±4.55^***^ | 32.79±4.74^***###^ | 32.17±5.08^***###$$^ | 31.86±5.07^***###$$^ | <0.001 |
|  | TMWM | 35.03±4.72 | 34.43±4.90^***^ | 33.70±4.50^***##^ | 33.14±4.89^***###$$^ | 33.09±4.82^***###$$^ | <0.001 |
| BFM (kg) | CIMWM | 32.03±6.32 | 29.77±6.15^***^ | 28.63±6.67^***###^ | 27.38±6.75^***###$$$^ | 27.41±6.88^***###$$^ | <0.001 |
|  | TMWM | 32.61±7.06 | 31.10±7.23^***^ | 29.59±6.19^***###^ | 28.94±6.46^***###$^ | 29.25±6.71^***###^ | <0.001 |
| FFM (kg) | CIMWM | 60.82±11.14 | 59.38±10.88^***^ | 58.10±10.10^***##^ | 56.97±10.34^***###$^ | 57.96±10.26^***##‡^ | <0.001 |
|  | TMWM | 60.30±9.88 | 58.86±9.31^***^ | 58.04±8.84^***#^ | 58.16±8.61^***^ | 58.25±8.90^***^ | <0.001 |
| SMM (kg) | CIMWM | 33.30±6.20 | 32.52±6.22^***^ | 32.01±5.44^***#^ | 32.22±6.87^**^ | 31.24±6.38^***##$^ | <0.001 |
|  | TMWM | 33.07±5.49 | 32.27±5.18^***^ | 31.85±4.92^***^ | 31.96±5.15^***^ | 31.59±5.35^***#^ | <0.001 |
| VFA (cm^2^) | CIMWM | 133.70±34.46 | 122.84±29.74^***^ | 116.70±32.11^***###^ | 111.32±34.29^***###$$$^ | 110.00±30.69^***###$$^ | <0.001 |
|  | TMWM | 140.46±48.59 | 133.62±45.31^**^ | 126.33±35.60^***#^ | 121.56±36.16^***###$^ | 120.01±32.76^***###$$^ | <0.001 |
| S/V (kg/cm^2^) | CIMWM | 0.26±0.08 | 0.27±0.07^**^ | 0.29±0.08^***##^ | 0.31±0.10^***###$$$^ | 0.30±0.10^***###$^ | <0.001 |
|  | TMWM | 0.27±0.23 | 0.26±0.06 | 0.27±0.06^#^ | 0.28±0.08^###$$^ | 0.28±0.07^###$$^ | <0.001 |
| WC (cm) | CIMWM | 103.59±11.78 | 101.30±10.17^**^ | 97.45±12.56^***###^ | 96.47±9.93^***###^ | 96.26±9.76^***###^ | <0.001 |
|  | TMWM | 103.70±12.74 | 102.36±10.06 | 99.52±9.07^***###^ | 98.35±9.79^***###^ | 98.01±8.74^***###$$^ | 0.001 |
| HC (cm) | CIMWM | 110.61±7.58 | 108.47±7.34^***^ | 106.27±7.90^***###^ | 104.90±8.71^***###$$^ | 105.65±7.62^***###^ | <0.001 |
|  | TMWM | 110.34±11.86 | 109.58±7.83 | 105.94±11.28^**###^ | 105.94±12.76^***###^ | 106.50±7.25^***###^ | <0.001 |
| WHR | CIMWM | 0.94±0.09 | 0.93±0.06 | 0.92±0.10*# | 0.92±0.07^*#^ | 0.91±0.05^***###^ | 0.002 |
|  | TMWM | 0.99±0.69 | 0.93±0.06 | 0.96±0.20 | 0.94±0.20 | 0.92±0.06^##^ | 0.007 |
| SBP (mmHg) | CIMWM | 130.88±12.79 | 125.95±11.90^***^ | 124.50±11.74^***^ | 123.77±11.64^***#^ | 124.22±10.49^***^ | <0.001 |
|  | TMWM | 131.73±13.64 | 127.25±13.51^***^ | 126.82±12.60^***^ | 124.52±11.09^***#$^ | 125.41±11.89^***^ | <0.001 |
| DBP (mmHg) | CIMWM | 82.66±10.22 | 78.04±8.90^***^ | 76.72±8.02^***^ | 76.42±7.82^***#^ | 77.02±7.31^***^ | <0.001 |
|  | TMWM | 83.75±10.70 | 79.41±9.48^***^ | 79.41±8.49^***^ | 78.02±9.02^***^ | 77.61±8.82^***#$^ | <0.001 |
| ALT (U/L) | CIMWM | 44.79±37.24 |  |  | 30.39±30.81^***^ | 29.93±29.71^***^ | <0.001 |
|  | TMWM | 38.36±26.57 |  |  | 27.75±17.04^***^ | 30.42±24.05^**^ | <0.001 |
| AST (U/L) | CIMWM | 27.98±14.08 |  |  | 21.66±11.59^***^ | 22.35±12.01^***^ | <0.001 |
|  | TMWM | 26.02±11.46 |  |  | 21.52±7.74^***^ | 23.54±13.50^‡^ | <0.001 |
| TC (mmol/L) | CIMWM | 5.15±1.05 |  |  | 4.72±0.81^***^ | 4.72±0.92^***^ | <0.001 |
|  | TMWM | 5.09±0.84 |  |  | 4.90±0.89^*^ | 4.93±0.86^*^ | 0.022 |
| TG (mmol/L) | CIMWM | 2.16±1.70 |  |  | 1.41±1.29^***^ | 1.69±1.10^**‡‡^ | <0.001 |
|  | TMWM | 1.98±1.12 |  |  | 1.38±0.66^***^ | 1.68±0.88^**‡‡‡^ | <0.001 |
| HDL-C (mmol/L) | CIMWM | 1.11±0.27 |  |  | 1.14±0.22 | 1.21±0.23^***‡‡‡^ | <0.001 |
|  | TMWM | 1.22±1.16 |  |  | 1.17±0.28 | 1.24±0.23^‡‡^ | 0.002 |
| LDL-C (mmol/L) | CIMWM | 3.19±0.74 |  |  | 3.02±0.67^**^ | 2.92±0.68^***^ | <0.001 |
|  | TMWM | 3.17±0.71 |  |  | 3.11±0.71 | 3.02±0.74^*^ | 0.022 |
| Lp (a) (mg/L) | CIMWM | 158.20±172.72 |  |  | 201.08±188.29^**^ | 206.11±227.23^**^ | 0.011 |
|  | TMWM | 140.05±145.57 |  |  | 189.20±158.38^***^ | 165.74±153.30^**‡^ | <0.001 |
| FPG (mmol/L) | CIMWM | 5.16±0.75 |  |  | 4.90±0.52^***^ | 4.93±0.91^**^ | 0.001 |
|  | TMWM | 5.13±1.08 |  |  | 4.93±0.62^*^ | 4.95±0.76 | 0.038 |
| Cr (µmol/L) | CIMWM | 70.25±15.70 |  |  | 68.95±14.63 | 69.64±14.42 | 0.341 |
|  | TMWM | 67.57±15.71 |  |  | 68.27±13.20 | 66.60±13.95 | 0.297 |
| UA (µmol/L) | CIMWM | 404.12±111.32 |  |  | 409.37±116.18 | 369.92±92.97^***###^ | <0.001 |
|  | TMWM | 389.11±116.72 |  |  | 385.62±93.74 | 370.00±83.57^*‡^ | 0.048 |
| FINS (pmol/L) | CIMWM | 157.82±198.46 |  |  | 89.87±42.22^***^ | 94.87±55.91^***^ | <0.001 |
|  | TMWM | 135.3±80.83 |  |  | 95.45±54.12^***^ | 103.08±56.22^***^ | <0.001 |
| FCP (pmol/L) | CIMWM | 1292.20±1201.69 |  |  | 906.44±489.83^**^ | 871.85±399.82^***^ | <0.001 |
|  | TMWM | 1062.05±454.12 |  |  | 941.64±648.49 | 864.65±277.24^***^ | <0.001 |
| HbA1c (%) | CIMWM | 5.51±0.42 |  |  | 5.34±0.33^***^ | 5.47±0.48^‡‡‡^ | <0.001 |
|  | TMWM | 5.48±0.37 |  |  | 5.39±0.33^**^ | 5.46±0.42^‡^ | 0.031 |
| IFG Number (%) | CIMWM | 11（8.9%） |  |  | 1（0.8%）^***^ | 2（1.6%）^***^ | <0.001 |
|  | TMWM | 10（7.6%） |  |  | 5（3.8%） | 9（6.9%） | 0.292 |
| PLT (×10^9^) | CIMWM | 267.80±56.57 |  |  | 253.61±55.56^***^ | 250.91±51.19^***^ | <0.001 |
|  | TMWM | 261.10±60.24 |  |  | 255.26±58.46 | 254.40±56.02 | 0.226 |
| ALB (g/L) | CIMWM | 45.90±4.26 |  |  | 46.25±4.07 | 46.75±3.99^**^ | 0.001 |
|  | TMWM | 45.48±3.96 |  |  | 45.30±4.56 | 46.06±2.56 | 0.142 |
| NFS | CIMWM | -3.12±1.01 |  |  | -3.10±0.99 | -3.13±0.85 | 0.901 |
|  | TMWM | -2.91±1.07 |  |  | -3.01±0.96 | -2.97±0.94 | 0.455 |
| Fibrosis Severity Scale |  |  |  |  |  |  |  |
| *F0-F2* | CIMWM | 119（96.7%） |  |  | 118（95.9%） | 119（96.7%） | 0.896 |
|  | TMWM | 121（92.4%） |  |  | 121（92.4%） | 121（92.4%） | 1 |
| *Indeterminant score* | CIMWM | 4（3.3%） |  |  | 5（4.1%） | 4（3.3%） | 0.896 |
|  | TMWM | 10（7.6%） |  |  | 10（7.6%） | 10（7.6%） | 1 |
| *F3-F4* | CIMWM | 0 |  |  | 0 | 0 |  |
|  | TMWM | 0 |  |  | 0 | 0 |  |
| HOMA-IR | CIMWM | 5.16±6.15 |  |  | 2.82±1.36^***^ | 2.30±1.89^***^ | <0.001 |
|  | TMWM | 4.42±2.78 |  |  | 3.05±1.93^***^ | 3.29±1.96^***^ | <0.001 |

*body mass index (BMI), body weight (BW), body fat percentage (BFP), body fat mass (BFM), fat-free mass (FFM), Skeletal muscle mass (SMM), visceral fat area (VFA), skeletal muscle mass-to-visceral fat area radio (S/V), waist circumference (WC), hip circumference (HC), waist-to-hip ratio (WHR), alanine aminotransferase (ALT), aspartate aminotransferase (AST), total cholesterol (TC), triglyceride (TG), high-density lipoprotein cholesterol (HDL⁃C), low-density lipoprotein cholesterol (LDL⁃C), Lipoprotein(a) [Lp(a)], creatinine (Cr), uric acid (UA), fasting plasma glucose (FPG), fasting insulin (FINS), fasting C peptide (FCP), platelet (PLT), albumin (ALB), glycated hemoglobin (HbA1c), non-alcoholic fatty liver disease score (NFS), impaired fasting glucose (IFG), systolic blood pressure (SBP), diastolic blood pressure (DBP), homeostasis model assessment of insulin resistance (HOMA-IR)*

*a. Data are represented as mean ± SD for continuous variables and as percentages for categorical variables.*

*b. Differences at each time point in two groups were analyzed using repeated measurement ANOVA; IFG Number and Fibrosis Severity Scale were analyzed using chi-square analysis.*

*c. Comparing 1, 2, 3, 6 months with baseline, ***p < 0.001, **p < 0.01 *p < 0.05; comparing 2, 3, 6 months with 1 month, ^###^p < 0.001 ^##^p < 0.01 ^#^p < 0.05; comparing 3, 6 months with 2 months,* *^$$$^p < 0.001 ^$$^p < 0.01 ^$^p < 0.05; comparing 3 months with 6 months, ^‡‡‡^p < 0.001 ^‡‡^p < 0.01 ^‡^p < 0.05*
